# INTERDEPENDENCY OF COUPLES’ EVERYDAY COGNITIVE PERFORMANCE

**DOI:** 10.1093/geroni/igae098.2691

**Published:** 2024-12-31

**Authors:** Martina Luchetti, Fiona Rupprecht, Jana Nikitin, Selin Karakose, Jeffrey Stokes, Antonio Terracciano, Angelina R Sutin

**Affiliations:** Florida State University, Tallahassee, Florida, United States; University of Vienna, Vienna, Wien, Austria; University of Vienna, Vienna, Wien, Austria; Florida State University, Tallahassee, Florida, United States; University of Massachusetts Boston, Boston, Massachusetts, United States; Florida State University, Tallahassee, Florida, United States; Florida State University College of Medicine, Tallahassee, Florida, United States

## Abstract

Longitudinal studies suggest an interdependency of marital partners’ well-being and health behaviors, which can impact cognitive health. To date, however, few studies have taken a dyadic approach to examine interrelations of cognitive function within romantic partners and none in daily life. This study examines momentary cognitive performance within middle-aged and older romantic couples, and how their performance is interrelated over 8 consecutive days. Data were from 137 opposite-sex couples (274 participants), between 40 and 70 years old, who completed a smartphone-based processing speed task and a visual memory task, three times a day, over the study period. A combination of dynamic structural equation and actor-partner interdependence modeling was used for the analysis. The analysis indicated that mean-level performance on both tasks were correlated between partners. There was a significant time-lagged partner effect for processing speed: Poor performance (longer reaction times) of a participant at one momentary assessment was associated with poor performance of their partner at the next assessment. When controlling for contextual factors (e.g., location, day of the week, presence/absence of others), only the time-lagged effect from female to male partners remained significant. Further, this time-lagged effect was stronger for couples of younger ages. There was no lagged effect for visual memory. This work sheds light on the interdependency of couples’ daily functioning, which might have implications for long-term cognitive outcomes.

